# From Our Correspondents

**Published:** 1982

**Authors:** 


### From our Correspondents

On the **surgical** front 1981/82 has had more than its fair share of notable events. The simultaneous retirements of Mr. Herbert Bourns (Infirmary/ Cossham) and Mr. Michael Wilson (Southmead) were marked by particularly grand Grand Rounds in June 1981. For this meeting the Saturday morning attendance was second to none, except perhaps for a similar occasion the following year (June 1982) held in honour of Mr. Robert Horton (Infirmary) and Professor John Mitchell (Infirmary). Silver bleeding bowls were presented, one to the vascular surgeon and the other to the master of surgical diathermy (avascular surgeon). With a nice sense of time, *The Times* announced John Mitchell's CBE the same day. Each of these bittersweet events took place after a gala dinner on the Friday night, and each protagonist was chairman and principal speaker at the subsequent scientific meeting.

1981 also saw the retirement of Mr. Norman Slade (Southmead) as a consultant urologist, and sadly in 1982 Mr. Douglas Mearns Milne (Frenchay) died within a year of his retirement as thoracic surgeon. A packed congregation for his memorial service in St.Mary Redcliffe included representatives of the business, football and medical communities of the City, members of St.John's Ambulance and the Lord Lieutenant of Avon.

The Royal College of Surgeons of England, with Sir Alan Parks in the van, came to Bristol on 11 March 1982 for the second time in three years. Some 15 members of Council heard two Hunterian Lectures at the Infirmary. Mr. W. E. G. Thomas celebrated his substantive appointment as senior surgical registrar by gaining an Arris and Gale Lectureship and the Moynihan Fellowship. Mr. Michael Kelly, another senior registrar, was BUPA Doctor of the Year. During 1982 Bristol provided a third Hunterian Professor and a second Arris and Gale Lecturer. Mr. Tony Ratliff was President of the British Orthopaedic Association, and Mr. J. B. M. Roberts joined the Court of Examiners at the College. The previous year Mr. R. W. Pigott won the Jacksonian Prize for his work on cleft palate.

A generous benefaction from the will of the late Mrs. Marjorie Budd enables the University Department of Surgery to endow an annual visiting professorship. The first Marjorie Budd professor was a distinguished scion of Bristol surgery, Professor Norman Browse of St. Thomas' Hospital, London. Centrepiece of his gruelling three-day programme in February 1982 was his magnificent inaugural lecture entitled 'Vascular surgery – art or science?' and delivered to an audience that included professor and Mrs. Robert Milnes Walker. Afterwards at a short ceremony the new reading room in the Department was baptised The Milnes Walker Room.

On the **university** front the medical faculty has developed florid thyrotoxicosis: querulous, over-excitable, losing weight and tremulous before impending cuts. Clinical medicine has not escaped the chill wind facing the rest of the University, despite Bristol costs of educating medical students being only 80 per cent of the national average.

Brighter spots in the olive grove of Academe have been new appointments to the professoriate: E. Rhys Davies (Radiodiagnosis) and Gordon Stirrat (Obstetrics and Gynaecology). David Easty has become first holder of the freshly-created Chair of Ophthalmology, and a Chair of Orthopaedic Surgery has been founded entirely by voluntary subscription. Most notable of all, Bristol received its first ever medical knighthood in 1981: Sir Howard Middlemiss, CMG, sometime Professor of Radiodiagnosis, Dean of the Faculty of Medicine and President of the Royal College of Radiologists.

R.C.N.W.

On 24th June, 1982, in the new Postgraduate Medical Centre at Southmead Hospital, a farewell dinner was held for Dr. Graham Airth, consultant radiologist to the hospital from 1950–1982. Dr. John Crossley when proposing Dr. and Mrs. Airth's health reminded the assembled company of friends and colleagues of the great part Dr. Airth played during his 32 years at Southmead in developing the radiological services both locally and throughout the Region. Dr. Airth was always in the van of his field and his close association with Phillips Electrical often enabled new developments to be 'tried out' at Southmead with benefits both to the clinicians and to the equipment budget. Dr. Airth in replying delighted and astonished his audience by donating to Southmead Hospital a copy of the English translation of Ambrose Parey's famous surgical treatise published in 1634 wishing that it should be kept in the hospital library and stressing that it should be available for study at any time.

Recently I decided to examine this marvellous gift. For an hour or so I sat in the library dipping lovingly into this original medical classic. It is entitled *The Workes of that famous Chirurgion*

*Ambrose Parey – Translated out of Latine and compared with the French* by Thomas Johnson in 1634. The original had been published in 1579 when Parey was Chief Chyrurgion to the King of France. It is impossible to describe the excitement of turning the 1032 pages of this stupendous work. Although the major part of the book is concerned with the surgical treatment of war wounds, there are long sections on anatomy, other surgical practice and virtually all aspects of medicine as seen in the 16th century. There are numerous illustrations and the section on orthopaedics is especially fascinating. The print of a Glossocomium (Yes – it is in the shorter OED!) or extender, for the traction treatment of a fracture of the thigh is not unlike some of the ironware seen today in a fracture ward. In the section on urology after a good description of renal colic the following treatment is suggested: 'Therefore let the patient, if he be able, mount upon a trotting horse, and ride upon him the space of two miles, or if he can have no opportunity to do so, then let him run up and down a paire of stairs until he be weary, and even sweat again: for the stone by this experience is oft times shaken into the bladder! This is gentle treatment in comparison to the effects which one could imagine were produced by a fearful series of instruments illustrated for lithotomy and lithotrity for bladder stones, which were very common in those times. Another item, 'Of the Diabete', or inability to hold the urine, caught my eye – but I must stop.

I hope I have whetted the appetite for all with the least interest in medical history. This lovely book has a wealth of diverse medical gems and must surely be consulted by anyone seeking for a 'starter' to a lecture or by the more serious student in most branches of medicine. If we cannot go back to an origin copy of Hippocrates all Southmead doctors will surely be quoting from Parey from now on.

Our thanks to Graham Airth for this magnificent volume will grow with the years and we are delighted to see him regularly in his retirement for lunch on Thursdays in the postgraduate centre where he is more than welcome to the 'old boys' table to which all the retired consultant staff of the hospital are cordially invited.

C.R.T.

**August 16th, 1982** – a notable day!

After this day all doctors entering general practice will have completed a three-year post registration period of training. Two of these years will have been in hospital posts and one year in a training practice.

**What is a training practice?** A training practice is one in which one of the principals has been appointed a trainer by the University Postgraduate Education Committee. To become this he must have attended a week's education course and be considered by three of his colleagues who inspect him and his practice to be suitable to become a trainer. He is expected to put time aside each week for teaching, to have a good record system and to be willing to put up with the disruption of a new young doctor each year. There are advantages of course: an extra pair of hands, extra income, new ideas and a doctor who investigates one aspect of the practice in detail each year – hopefully, not finding too many skeletons in the cupboard!

M.J.W.

Frenchay Hospital was sad to loose one of their Senior Radiologists earlier this year. Dr. David McKenzie-Crooks left the department to take up the post of director in one of the main hospitals in Riyadh, Saudi Arabia.

At a farewell party, several useful gifts were presented, including a flute (he is already an accomplished musician) for amusement after hours, and a plastic arm as a replacement prosthesis should any cause be found to remove the original. One of the radiographers was suitably dressed as an Arab with a trolley covered in a white sheet from which to make the presentation.

The hospital now welcomes Dr. Andrew Longstaff as a replacement, ex-Navy and recently Sheffield University X-Ray department trained.

G.T.

Dr. Ronnie Walley, Consultant in Infectious Diseases at Ham Green Hospital retired in February 1982 after 29 years at the hospital. Tributes were paid to his many talents outside Medicine and it was fitting that he should choose a lathe as his retirement present. This was suitably inscribed PHYSICIAN AND CRAFTSMAN. He will also now have time to enjoy his other 'love' motor cycling.

Dr. Kay Iles, having spent most of her professional life working at Ham Green Hospital and the Chest Clinics, died suddenly whilst still working in October, 1980. Her family and friends decided a lasting memorial to all the care she gave to her paients should be established the form of a memorial garden which has been developed near the chapel in the hospital, the cost of which has come from donations from her many friends and patients. The garden is now nearing completion and a plaque in commemoration has been erected on the chapel wall. We expect many of her friends will come to visit it and already it is being widely used by both staff and patients.

During this year we have been joined by three new consultants:—

Dr. Stuart Glover, who took over from Dr. R. V. Walley, in Infectious Diseases and who is already taking a very active part in post- and undergraduate teaching.

Dr. Peter Hollingsworth, Consultant Rheumatologist, who has some beds at Ham Green and is a very welcome addition to our Staff.

Dr. William Colwell, Consultant Geriatrician who is a much needed colleague for Dr. Geoffrey Burston who, until now, has managed single-handed.

D.W.W.

Our thoughts at the moment are very much centred on the financial plight of the N.H.S. in general and in Bristol in particular.

Under such introspective circumstances it is always useful to obtain a new perspective, such as that provided for me by Peter Bewes in his Personal view (*B.M.J.* 285, p. 729, 1982). He has returned to the U.K. after six years as a surgeon in a 320-bed hospital in Northern Tanzania. The problem he and his colleagues had to face was cut by 40% in the financial allocation to the hospital. If the saving were not made on materials and consumables, it would come from staff salaries. Did they manage it? Yes, they did, and these are among the ways they achieved it. 'Disposable' items were re-used time and time again. Blades and needles were re-sharpened. Indwelling catheters were abolished. Dressings were simplified. Prescribing freedom was drastically curtailed. The cost of suture materials fell to one-third of the previous cost. As a result, for example, the incidence of wound and urinary infections fell and fractures seemed to heal more quickly.

Thus did one group of doctors achieve "a clearer understanding of surgery and of their proper priorities".

It makes you think, doesn't it?

